# Correlation of periodontal diseases with intracranial aneurysm formation: novel predictive indicators

**DOI:** 10.1186/s41016-021-00249-x

**Published:** 2021-06-07

**Authors:** Keyun Liu, Jia Sun, Lingling Shao, Hongwei He, Qinglin Liu, Youxiang Li, Huijian Ge

**Affiliations:** 1 Department of Interventional Neuroradiology, Beijing Neurosurgical Institute and Beijing Tiantan Hospital, Capital Medical University, No. 119, West Road of South Fourth Ring, Fengtai, Beijing, 100070 People’s Republic of China; 2 Department of Stomatology, Tianjin Stomatological Hospital, Hospital of Stomatology, Nankai University, Tianjin, 300041 People’s Republic of China; 3 Department of Tuberculosis, Beijing Tuberculosis and Thoracic Tumor Institute, Beijing Chest Hospital, Capital Medical University, Beijing, 101149 People’s Republic of China

**Keywords:** Gingivitis, Intracranial aneurysms, Inflammation, Periodontitis

## Abstract

**Background:**

We investigated whether periodontal diseases, specifically, periodontitis and gingivitis, could be risk factors of the incidence of intracranial aneurysms (IAs).

**Methods:**

We performed a case–control study to compare the differences in the periodontal disease parameters of 281 cases that were divided into the IAs group and non-IAs group. All cases underwent complete radiographic examination for IAs and examination for periodontal health.

**Results:**

Comparing with those in the non-IAs group, the cases in the IAs group were older (53.95 ± 8.56 vs 47.79 ± 12.33, p < 0.001) and had a higher incidence of hypertension (76 vs 34, p = 0.006). Univariate logistic regression analysis revealed that age (> 50 years) and hypertension were predictive risk factors of aneurysm formation (odds ratio [OR] 1.047, 95% confidence interval [95% CI] 1.022–1.073, p < 0.001 and OR 2.047, 95% CI 1.232–3.401, p = 0.006). In addition, univariate and multivariate logistic regression analyses showed that the parameters of periodontal diseases, including gingival index, plaque index, clinical attachment loss, and alveolar bone loss, were significantly associated with the occurrence of IAs (all p < 0.05). For further statistical investigation, the parameters of periodontal diseases were divided into four layers based on the quartered data. Poorer periodontal health condition (especially gingival index > 1.1 and plaque index > 1.5) had the correlation with IAs formation (p = 0.007 and p < 0.001).

**Conclusion:**

Severe gingivitis or periodontitis, combining with hypertension, is significantly associated with the incidence of IAs.

## Background

Intracranial aneurysms (IAs) are pathological dilatations of cerebral arteries; they are most often saccular in shape and frequently found in proximal cerebral artery bifurcations [1]. Subarachnoid hemorrhage caused by IA rupture affects 10–11/100,000 population per year in Western populations [2]. IAs rupture and subsequent hemorrhage may account for a mortality rate of 35%, and most survivors are left with considerable neurological impairment [3, 4]. Unruptured IAs are commonly treated through endovascular intervention or neurosurgical procedures to decrease the possibility of subarachnoid hemorrhage. However, most unruptured IAs are asymptomatic, and patients ignore potential risks. Thus, IAs must be diagnosed accurately before rupture, and the pathological mechanism of aneurysm formation should be emphasized.

Periodontitis and gingivitis are chronic inflammatory condition leading to the irreversible destruction of the tooth supporting tissues (gingiva, periodontal ligaments, and alveolar bone) with tooth loss as a common end point [5–7]. Periodontitis results from the complex pathological reactivity between chronic bacterial infection and host inflammatory response [8]. Gingivitis is defined as the inflammatory injury in the gingiva that are most commonly induced by the accumulation of dental plaque. In recent years, references have investigated the correlation between systematic vascular diseases and periodontitis or gingivitis. Lockhart et al. reported that oral bacterial deoxyribonucleic acid (DNA) had been extracted in peripheral arteries, and suggested that oral infections might contribute to vascular wall inflammation [9]. Bacteria from periodontal pockets and their secreta, such as endotoxins, have been identified in cardiovascular atherosclerotic lesions [10–12]. Iwai investigated periodontal bacterial DNA in abdominal aortic aneurysmal walls and then concluded that poor periodontal conditions have important effects on the progression of abdominal aortic aneurysms [13].

Additional nine studies confirmed the significant correlation between periodontal diseases and cerebrovascular strokes [14–22]. Further clinical studies speculated that periodontal inflammation caused by Streptococcus mutans participates in the occurrence of intracranial hemorrhage strokes [23–25]. However, evidence for the role of periodontal diseases in IAs was insufficient because of the small sample sizes. Therefore, we performed this case–control study to investigate whether periodontal diseases were associated with IA formation and tried to discover novel epidemiological evidence.

## Method

### Study population

From September 2015 to September 2018, we collected cases that were admitted to our institution for IAs diagnosis via digital subtraction angiography (DSA) or computer tomography angiography (CTA) examination. During the same period, we also recruited cases that were diagnosed without IAs or clinical trial volunteers. A total of 281 cases signed written informed consent and were divided into two groups (166 cases in the IAs group and 115 cases in the non-IAs group). The privacy of the patients was strictly protected, and the protocol of this study was approved by our ethics committee. Periodontal health examination were provided to all recruited cases through a standardized approach in a dental unit by using a standard dental light, compressed air, a mouth mirror, and digital panoramic radiography free of charge. Periodontal disease was diagnosed on the basis of the clinical and radiographic criteria described by the 1999 consensus classification of periodontal diseases [26].

Inclusion criteria were as follows: (1) individuals who were 18–80 years of age, (2) individuals with IAs and hospitalized volunteers (non-IAs) who wanted to participate in this study, and (3) individuals who underwent the examination of periodontal health condition. Exclusion criteria included (1) individuals with acute ruptured aneurysms; (2) individuals receiving antihypertensive therapy with calcium channel blockers, such as nifedipin; (3) individuals with severe cardiovascular diseases or cerebral ischemic stroke; (4) individuals with malignant diseases, chronic inflammatory diseases, or antibiotic use within 2 weeks; and (5) individuals missing clinical follow-up. Baseline demographic information (including age, sex, clinical presentation, cerebrovascular history, smoking and drinking history, diabetes, hypertension, and hypercholesterolemia) and IAs characteristics (location, size, shape, and quantity) were recorded.

### Assessment of periodontal diseases

Periodontal diseases mainly included periodontitis and gingivitis. Gingivitis severity was assessed by using the gingival index (GI) system based on the various tendencies of gingival bleeding after gingival irritation. A high index number (> 1.1) was defined as severe gingivitis. Periodontitis was evaluated by using the parameters plaque index (PI), clinical attachment loss (CAL), and alveolar bone loss (ABL). Plaque and gingivitis were scored at four sites per tooth (buccal, mesiolingual, lingual, and distolingual) and averaged for each subject. We presented these parameters in accordance with the following definitions: first, the CAL was examined by inserting the tip of a community periodontal index (CPI) probe to measure the distance between the pocket and cementoenamel junction. Attachment levels were analyzed as continuous variables, and mean CAL > 4 mm was considered as severe periodontitis. Second, dental plaque was scored in accordance with the PI system, which is based on the same principle as the GI system. We divided PI into 4 sites (≤ 0.5, 0.51–1.0, 1.01–1.5, and > 1.5). A high score represented severe periodontitis. Third, ABL levels were measured as the distance from the cementoenamel junction to the most apical extension of the bony defect. We stratified the ABL into < 3.00, 3.00–4.00, 4.00–5.00, and > 5.00 mm.

### Statistical analysis

Data were presented as mean ± standard deviation or expressed in terms of frequencies and percentages. Independent sample t test and chi-square test were performed for differences in continuous or categorical variables between the IAs group and the non-IAs group, respectively. Subgroup univariate and multivariate logistic regression analyses were performed to identify the independent contribution of gingivitis and periodontitis parameters (including GI, CAL, PI, and ABL) to the incidence of IAs. Odds ratios (ORs) and 95% confidence intervals (CIs) were given for all periodontal parameters. A two-sided p value of < 0.05 was considered to be significant. Statistical analysis was performed by using the SPSS 22.0 software (SPSS Inc., Chicago, IL, USA).

## Results

A total of 281 cases (including 174 [61.9%] females and 107 [38.1%] males) were recruited in this study. The age ranged from 18 years to 79 years (51.34 ± 10.56 years). Table 1 showed the baseline characteristics and subgroup analysis results of the IAs group and non-IAs group. There were no statistically significant differences in the distributions of sex, diabetes mellitus, hyperlipidemia, cerebrovascular history (including cerebral ischemic and hemorrhage events), other personal history (such as alcohol, smoking, and BMI) in the two subgroups (all p > 0.05). Chi-square tests indicated that cases in the IAs group were older than those in the non-IAs group (53.95 ± 8.56 vs 47.79 ± 12.33, p < 0.001) and had a higher incidence of hypertension (76 vs 34, p = 0.006).

**Table 1:** Baseline characteristics of patients

Variable	Total, n = 281	IA(+), n = 166	IA(−), n = 115	P value (IA+ vs IA−)
Mean age (years)	51.34 ± 10.56	53.95 ± 8.56	47.79 ± 12.33	< 0.001
Female sex, n (%)	174 (61.9)	99 (59.6)	75 (65.2)	0.383
Hypertension, n (%)	110 (39.1)	76 (45.8)	34 (29.6)	0.006
Diabetes mellitus, n (%)	26 (9.3)	11 (6.6)	15 (13.0)	0.093
Hyperlipidemia, n (%)	26 (9.3)	17 (10.2)	9 (7.8)	0.537
Cerebrovascular diseases history, n (%)	18 (6.4)	10 (6.0)	8 (6.9)	0.807
Heavy smoking history, n (%)	54 (19.2)	36 (21.7)	18 (15.7)	0.222
Bibulosity, n (%)	39 (13.9)	19 (11.4)	20 (17.4)	0.165
Family history of cerebrovascular diseases, n (%)	30 (10.7)	21 (12.7)	9 (7.8)	0.241
Mean BMI	24.89 ± 4.06	25.18 ± 4.25	24.46 ± 3.77	0.145

Data are shown as mean ± SD or absolute and chi-square test between 2 groups

*BMI* body mass index

Cerebrovascular diseases history was defined as the patients who presented with cerebral ischemic and hemorrhage before inpatient

Univariate regression analysis revealed that aged cases (over 50 years) combining with these periodontal parameters and hypertension, were significantly associated with the occurrence of IAs (all p < 0.05) (Table 2). The parameters of periodontal diseases (GI, CAL, PI, and ABL) were divided into four layers on the basis of their averages and referenced literature [27]. Table 3 demonstrated that severe periodontitis and gingivitis might be accompanied by the higher risk of IAs formation. GI could be considered as a risk predictive factor of IAs (OR of GI > 1.1 17.11, 95% CI 3.339–87.66, p = 0.001). Higher PI (mainly > 1.5) showed the similar correlation (OR 6.968, 95% CI 2.396–20.259, p < 0.001). High CAL (> 4.00 mm), which indicated periodontitis with increased severity, was accompanied by the high risk of IA (OR 4.074, 95% CI 1.012–16.391, p = 0.048). The severity of ABL (> 4.00 mm) represented another significant risk factor of IAs with two OR values, specifically, 4.00–5.00 mm (p = 0.003), which corresponded to an OR of 6.409, and over 5.0 mm (p = 0.005), which corresponded to an OR of 21.835. The values of 95% CI are shown in Table 3. All of the above parameters of severe periodontal diseases had p < 0.05. While comparing with parameters CAL and ABL, the severe parameters PI (> 1.5) and GI (> 1.1) were associated with increased diagnostic value of aneurysms formation according to multivariate logistic regression analyses (Table 4, p < 0.001 and p = 0.007).

**Table 2:** Univariate logistic regression analysis of clinical characteristics and periodontitis parameters for intracranial aneurysm formation

Variables	Univariate regression analysis			
	P value	OR	95% CI	
Age (> 50 years)	< 0.001	1.047	1.022	1.073
Hypertension	0.006	2.047	1.232	3.401
GI	< 0.001	11.428	5.318	24.557
PI	< 0.001	3.053	1.908	4.885
CAL	0.001	1.794	1.252	2.571
ABL	< 0.001	2.211	1.613	3.030

*OR*, odds ratio; *95% CI*, 95% confidence interval; *GI*, gingival index; *PI*, plaque index; *CAL*, clinical attachment loss; *ABL*, alveolar bone loss

**Table 3:** Univariate logistic regression analysis for the correlation between four layers of periodontitis parameters and intracranial aneurysm

Variable		OR	95% CI		P value
			Lower limit	Upper limit	
GI	< 0.3 ^a^				
	0.3-0.7	0.553	0.258	1.185	0.128
	0.71-1.1	2.034	0.844	4.905	0.114
	> 1.1	17.110	3.339	87.660	0.001
PI	≤ 0.5 ^b^				
	0.51-1.0	0.687	0.341	1.384	0.294
	1.01-1.5	0.852	0.349	2.084	0.726
	> 1.5	6.968	2.396	20.259	< 0.001
CAL	< 3 mm ^c^				
	3.00-3.50 mm	1.001	0.509	1.970	0.997
	3.50-4.00 mm	0.433	0.176	1.061	0.067
	> 4.00 mm	4.074	1.012	16.391	0.048
ABL	< 3 mm ^d^				
	3.00-4.00 mm	1.410	0.775	2.564	0.260
	4.00-5.00 mm	6.409	1.907	21.533	0.003
	> 5.00 mm	21.835	2.503	190.463	0.005

Adjusted with age, hypertension

*OR*, odds ratio; *95% CI*, 95% confidence interval; *GI*, gingival index; *PI*, plaque index; *CAL*, clinical attachment loss; *ABL*, alveolar bone loss

^a^“<0.3” used as reference group in the binary logistic regression

^b^“≤0.5” used as reference group in the binary logistic regression

^c^“<3mm” used as reference group in the binary logistic regression

^d^“<3mm” used as reference group in the binary logistic regression

**Table 4:** Multiple logistic regression analysis for the correlation between severe periodontitis parameters and intracranial aneurysm

Variable	OR	95% CI		P value
		Lower limit	Upper limit	
GI > 1.1	8.001	1.751	36.557	0.007
PI > 1.5	6.438	2.381	17.406	< 0.001
CAL > 4.00 mm	2.360	0.600	9.287	0.219
ABL > 4.00 mm	7.989	0.958	66.600	0.055

*OR*, odds ratio; *95% CI*, 95% confidence interval; *GI*, gingival index; *PI*, plaque index; *CAL*, clinical attachment loss; *ABL*, alveolar bone loss

## Discussion

Periodontitis and gingivitis are associated with the risk of several diseases, such as rheumatoid arthritis and atherosclerosis [28, 29]. Recent studies have discussed the role of periodontal diseases in causing cerebral ischemia. Chiu demonstrated that periodontitis was associated with stroke which commonly caused by large-artery atherosclerosis; this result supported the hypothesis of a possible link between periodontitis and atherosclerosis [30]. After adjusting for confounding vascular factors on the basis of etiologic subgroup analysis, Grau et al. concluded that severe periodontitis was an independent risk predictive factor for IAs with atherothrombotic origins (OR 2.35 [1.00–11.0] and OR 13.2 [2.68–64.7]) and that gingivitis was independently associated with cerebral ischemia given its value as an indicator of the actual status of periodontal inflammation [31]. These findings suggested that chronic periodontal inflammatory responses contributed to a prothrombotic state via recurrent bacteremia and platelet or endothelial activation. Plaque destabilization is a potential trigger of cardioembolism and cryptogenic stroke [32, 33]. In addition, systemic immunization induced by local periodontitis may influence the accumulation of oxidized lipids in vascular walls and subsequent atherosclerotic remodeling indirectly.

Periodontal diseases, especially periodontitis, are associated with the remodeling of the aneurysmal wall in abdominal aortic aneurysms. In experimental models, periodontal bacteria promote the degeneration of the abdominal aortic aneurysmal wall by increasing the recruitment of neutrophils to the intraluminal thrombus that covers the inner portion of the abdominal aortic aneurysm [34]. The pathology of IA and abdominal aortic aneurysm shares several features [35]. The current understanding is that IAs formation as the end result of flow-driven inflammatory cell-mediated cerebral artery wall remodeling at sites where high flow exerts high wall shear stress [1]. However, IA is not detected in all cases under high flow and shear stress in the bifurcations of cerebral arteries. Previous studies on the correlation between periodontal diseases and stroke focused on the pathological mechanism of IA. Pyysalo detected the presence of oral bacteria DNA in ruptured and unruptured intracranial aneurysmal walls firstly [36]. Hallikainen et al. speculated that periodontitis predisposes the artery wall toward aneurysm development [37]. However, few works have paid attention to the relationship between intracranial hemorrhagic diseases and periodontal diseases. In this case–control study, we investigated the potential correlation between periodontal diseases (including periodontitis and gingivitis) and IAs formation. Although GI, PI, CAL, and ABL have long been known as actual indicators of periodontal inflammation caused by multitudinous oral bacteria, we considered that these parameters were independent predictive risk factors of IAs formation firstly. Further subgroup analysis demonstrated that severe periodontitis parameters might result in the high incidence of IAs. A past observation discussed that systemic elastase activity might play an important role given that increased serum elastase concentrations were associated with IAs, although the source of serum elastase was unknown [38]. Potential sources for circulating elastase were macrophages or neutrophils [39]. Tooth brushing or chewing could disseminate periodontal porphyromonas gingivalis (PG), especially in patients with periodontitis or gingivitis, to extraoral sites via circulation and then induce systemic inflammatory responses through transient bacteremia [40]. PG is available for modifying dendritic cell function and cause proinflammatory cytokine production in macrophages. Thus, periodontal pathogens that infiltrating cerebral arteries likely lead to excessive collagen degradation and neutrophil accumulation in the thrombus, promoting changes in the course of cerebral artery remodeling [41]. In another mechanism, neutrophils induced by periodontal pathogens are a major source of proteolytic activity because they release the matrix metalloproteinases 8 (MMP-8) and MMP-9, myeloperoxidase, and elastase and then accelerate the course of proteolytic or cytotoxic injury [27]. The ultimate pathology is damage to the elastic fibers of smooth muscles and the degeneration or necrosis of the intima medium. In combination with chronic hypertension, external vascular walls swell to form IAs.

Previous literature has indicated that aged cases (especially over 50 years) and hypertension are risk predictive factors of IAs formation [42, 43]. In the present study, we included patients who were older than 50 years or suffered hypertension and found that these cases had an increased incidence of IAs. Systemic hypertension not only affects tissue remodeling or vascular wall inflammation by exerting abnormal hemodynamic stresses but also activates the local renin–angiotensin system [44]. Moreover, hypertension may mediate vascular inflammation through the activation of NF-kappa B, which can further promote inflammation [45]. The continuous stimulatory response caused by hypertension and inflammation can result in the degeneration of vascular walls and subsequent IA formation.

This study has several limitations. First, although the literature has indicated that periodontal treatment might induce bacteria to transfer to systematic circulation, we could not persuade all patients to undergo periodontal or caries treatment because of their poor obedience. Thus, we will further study whether periodontal treatment has low relevance to IA. Second, the specific values of periodontal disease parameters that have the optimal predictive values for the incidence of IAs should be quantified through receiver operating characteristic curve analysis. Third, we did not perform further experimental research on inflammatory mediators that might cause IA directly. Fourth, other studies have demonstrated that age and hypertension were associated with the high risk of periodontal diseases. In this study, we only attempted to investigate the mechanism of aneurysm formation and neglected the relationship between these two factors and periodontal diseases.

## Conclusions

Periodontal diseases are significantly associated with the high incidence of IA. Given our results, patients who suffer from severe gingivitis or periodontitis and hypertension should be encouraged to undergo cerebrovascular examination. Mechanistic experiments on inflammatory response will positively predict the risk of IAs formation.

## Data Availability

The datasets used and/or analyzed during the current study are available from the corresponding author on reasonable request.

## Abbreviations

ABL: Alveolar bone loss
BMI: Body mass index
CAL: Clinical attachment loss
CIs: Confidence intervals
CPI: Community periodontal index
CTA: Computer tomography angiography
DNA: Deoxyribonucleic acid
DSA: Digital subtraction angiography
GI: Gingival index
IA: Intracranial aneurysm
MMP: Matrix metalloproteinases
OR: Odds ratios
PI: Plaque index
PG: Porphyromonas gingivalis
SAH: Subarachnoid hemorrhage
